# Reverting *TP53* Mutation in Breast Cancer Cells: Prime Editing Workflow and Technical Considerations

**DOI:** 10.3390/cells11101612

**Published:** 2022-05-11

**Authors:** Asmaa Y. Abuhamad, Nurul Nadia Mohamad Zamberi, Ling Sheen, Safaa M. Naes, Siti Nur Hasanah Mohd Yusuf, Asilah Ahmad Tajudin, M. Aiman Mohtar, Amir Syahir Amir Hamzah, Saiful Effendi Syafruddin

**Affiliations:** 1 Nanobiotechnology Research Group, Department of Biochemistry, Faculty of Biotechnology and Biomolecular Sciences, University Putra Malaysia, Serdang 43400, Selangor, Malaysia; gs56789@student.upm.edu.my (A.Y.A.); asilah_at@upm.edu.my (A.A.T.); 2 UKM Medical Molecular Biology Institute, University Kebangsaan Malaysia, Jalan Yaacob Latiff, Bandar Tun Razak, Kuala Lumpur 56000, Malaysia; sakinahjz@gmail.com (N.N.M.Z.); ling_sheen@yahoo.com (L.S.); hasanahyusuf@ukm.edu.my (S.N.H.M.Y.); m.aimanmohtar@ppukm.ukm.edu.my (M.A.M.); 3 Department of Biochemistry & Molecular Medicine, Faculty of Medicine, University Teknologi MARA, Cawangan Selangor, Kampus Sungai Buloh, Sungai Buloh 47000, Selangor, Malaysia; safanaes@gmail.com; 4 Department of Microbiology, Faculty of Biotechnology and Biomolecular Sciences, University Putra Malaysia, Serdang 43400, Selangor, Malaysia; 5 UPM-MAKNA Cancer Research Laboratory, Institute of Bioscience, University Putra Malaysia, Serdang 43400, Selangor, Malaysia

**Keywords:** prime editing, CRISPR, TP53, missense mutation, tumor suppressor, breast cancer

## Abstract

Breast cancer is the leading cause of cancer-related deaths in women. The aggressive breast cancer subtype is commonly linked to the genetic alterations in the *TP53* tumor suppressor gene, predominantly the missense mutations. Robust experimental models are needed to gain better insights into these mutations’ molecular properties and implications in tumorigenesis. The generation of such models harboring the alterations is feasible with the CRISPR-based gene editing technology. Moreover, the development of new CRISPR applications, particularly DNA base and prime editing, has considerably improved the precision and versatility of gene editing. Here, we employed the prime editing tool to revert a *TP53* missense C > T mutation (L194F) in a T47D luminal A breast cancer cell line. In parallel, this prime editing tool was also utilized to introduce the L194F mutation in HEK293T cells. To assess the prime editing efficiency in both cell lines, we first performed Sanger sequencing in the prime-edited cells pool and single cell-derived clones. However, the Sanger sequencing approach did not detect any base substitution in these cell lines. Next, by employing the more sensitive amplicon target sequencing, we managed to identify the expected substitution in these T47D and HEK293T cells, albeit the editing efficiency was low. In light of these findings, we discussed the technical aspects and provided suggestions for improve the prime editing workflow and efficiency for future experiments.

## 1. Introduction

In women, as of 2020, breast cancer was the most-diagnosed cancer and the leading cause of cancer-related death [1]. Advancements in the early detection tools and development of novel treatment strategies have largely improved the patients’ prognosis [2,3]. Nonetheless, the survival rate of breast cancer patients, especially those with metastatic disease, is still low [4,5]. Moreover, the widespread inter- and intratumoral heterogeneities have also contributed to the unsatisfactory treatment response in a significant fraction of breast cancer patients, subsequently leading to aggressive and incurable disease [6,7,8]. Hence, in order to improve the patients’ treatment response, the therapeutic strategies need to be personalized according to the patient mutations profile or genetic background. This personalized medicine approach has been made possible by the global efforts to comprehensively map the breast cancer molecular landscape, and this knowledge could pave the way for the development of efficient therapeutic strategies [9,10,11,12,13].

According to The Cancer Genome Atlas (TCGA), *TP53* was among the most frequently altered genes in their breast cancer patient cohort [9,11]. On top of this, the *TP53* is also the most commonly mutated gene across human cancers [14,15]. Notably, the *TP53* mutation hotspot in human cancers is located within the DNA binding domain (DBD) [15]. In breast cancer, an increase in the *TP53* mutations burden positively correlated with advanced disease and poor clinical outcomes [16,17,18,19]. Deemed as the guardian of the genome, the transcription factor TP53 plays vital roles in controlling cell division and preserving the genome stability and integrity [20]. In the event of DNA damage, TP53 activates signaling cascades to halt the cell cycle progression; this is followed by either repairing the damage or inducing cell apoptosis if the damage is irreparable [20]. Playing a predominant role as a tumor suppressor, mutations in *TP53* (mutTP53) would result in the loss-of-functions that would, in turn, promote tumorigenesis [21]. Interestingly, mutTP53 could also acquire an oncogenic gain-of-functions, or exert dominant negative effects over the tumor suppressive wild-type TP53 [21,22]. Regardless of whether there is a loss- or gain-of-functions, mutations in *TP53* would lead to genomic instability, promote cancer cell growth and survival, increase metastatic capabilities, and confer therapeutic resistances. Better insights into these mutant TP53 properties and phenotypic effects in cancer are important to facilitate the development of efficient therapeutic strategies.

Rapid advancements in the CRISPR-Cas9 gene editing technology, which was originally repurposed from the bacterial adaptive immune system, has endowed researchers with a robust toolbox for studying gene functions and regulations [23,24,25]. These include the capabilities to perform gene knockout, gene knock-in, transcription regulation modulation, epigenetic modifications, and chromosome visualizations using either the wild-type Cas9 or the engineered Cas9 variants [26,27]. Additionally, several CRISPR-Cas9-based therapies have been either clinically evaluated or are currently undergoing trials for treating cancer and monogenic diseases, such as B-Thalassemia, sickle cell disease, and vision loss [28,29,30,31]. Nonetheless, the efficacy and safety issues related to double-strand break (DSB) and the off-target effects have remained the major concerns, hence hindering the full exploitation of this gene editing technology for therapeutic uses [32]. The advent of novel CRISPR-Cas9-based applications, namely base editing and prime editing, has further advanced the gene editing field [33,34,35]. These latest technologies do not require a DSB and repair template for precise editing, therefore overcoming the traditional CRISPR-Cas9 gene editing technology limitations [36].

Prime editing is deemed to offer superior editing efficiency and versatility by which it can edit almost any DNA base, as well as introduce small insertion/deletions (INDELs) [37]. The prime editing approach relies on prime editing gRNA (pegRNA) and the reverse transcriptase (RT) enzyme, which is fused to the Cas9 nickase (Cas9n), to introduce the desired edits [35]. The RT repair template and primer binding site (PBS) is incorporated into the pegRNA, which is also composed of a pegRNA spacer that functions to guide the RT-fused Cas9n to the target region [35]. Although it was just recently developed, there are already a growing number of studies employing this prime editing tool in different experimental models [38,39,40,41,42,43,44], hoping that it could be further developed and approved for future clinical uses. In this present study, we aimed to assess the efficiency and establish a prime editing workflow by reverting a *TP53* missense mutation in the T47D luminal A breast cancer cell line (c.580 C > T, p.L194F). Additionally, we also utilized the prime editing tool to introduce the aforementioned *TP53* mutation in HEK293T cells. For the positive control, we attempted to substitute a base (C > G) using this prime editing tool in the previously reported HEK3 region [35,40]. For the *TP53* prime editing, the Sanger sequencing approach was not able to detect the expected edits either in the T47D or HEK293T cell pools and their single cell-derived clones. Nevertheless, we managed to identify the respective *TP53* edits in T47D and HEK293T cell pools using the more robust and sensitive amplicon target sequencing approach. By utilizing the same approach, we were also able to detect the desired edits in the HEK3 region in the T47D and HEK293T cell pools. Even though there were prime editing events in these cells, it is important to note that the efficiencies were low. Therefore, we also discussed the technical considerations for improving the prime editing efficiency, positing ways to move forward that could be beneficial for researchers in this field.

## 2. Materials and Methods

### 2.1. Cell Lines and Reagents

The T47D human luminal A breast cancer cells (ATCC HTB-133), MDA-MB-231 human triple-negative breast cancer cells (ATCC HTB-26), and HEK293T cells were obtained from the American Type Culture Collection (ATCC, Manassas, VA, USA). These cell lines were confirmed to be mycoplasma negative by using the e-Myco^TM^ Mycoplasma PCR Detection Kit (Intron, Kirkland, WA, USA). The identity of the T47D cell line was validated using Sanger sequencing-based detection of the known *TP53* mutation that is unique to this cell line, as annotated in the Sanger Institute (https://cancer.sanger.ac.uk/) and Ensembl (http://www.ensembl.org/) databases (both accessed on 1 September 2020). All these three cell lines were cultured in DMEM (Nacalai Tesque, Kyoto, Japan) supplemented with 10% heat-inactivated FBS (Tico Europe, Amstelveen, The Netherlands) and 1% penicillin-streptomycin solution (Nacalai Tesque). 

The pCMV-PE2 (Addgene plasmid #132775) and pU6-pegRNA-GG-acceptor (Addgene plasmid #132777) were gifts from David Liu [35]. The pKLV-U6-gRNA(BbsI)-PGKHygro2AeGFP was previously generated by Syafruddin et al. [45], which was modified from the pKLV-U6-gRNA(BbsI)-PGKpuro2ABFP sgRNA expression vector [46]. The packaging plasmids psPAX2 (Addgene plasmid #12260) and pMD2.G (Addgene plasmid #122259) were gifts from Didier Trono. The *TP53* T > C pegRNA, *TP53* C > T pegRNA and *HEK3* C > G pegRNA were purchased from Eurofins. The *TP53* and *HEK3* nicking sgRNAs (PE3) and all primers used in this study were purchased from Integrated DNA Technologies (IDT). These sequences are available in Supplementary Tables S1 and S2. 

### 2.2. pegRNAs and Nicking sgRNA Plasmid Construction (PE3)

Each pegRNA was designed to harbor BsaI restriction sites at the 5′ and 3′ end. The single-stranded pegRNAs were amplified to produce double-stranded pegRNAs using Accuprime PFX Supermix, according to the manufacturer’s recommendation (Invitrogen, Waltham, MA, USA). The amplified pegRNAs were digested with BsaI-HF restriction enzyme (New England Biolabs, Ipswich, MA, USA) and ligated into Antarctic phosphatase-treated/BsaI-digested pU6-pegRNA-GG-acceptor plasmid. For constructing the PE3 plasmids, the complementary top and bottom sgRNA strands were purchased separately. Each strand was designed to harbor the BbsI restriction site at the 5**′** ends. The 5′ end of each strand was phosphorylated by using the T4 polynucleotide kinase (New England Biolabs) and annealed together. The annealed PE3 was ligated into Antarctic phosphatase-treated/BbsI-digested pKLV-U6-gRNA(BbsI)-PGKHygro2AeGFP. Briefly, the ligation was performed using the T4 ligase (New England Biolabs) at 16 °C overnight, followed by transformation into the chemically competent DH5α E.coli strain (New England Biolabs). The presence of ligated pegRNA and PE3 into their respective expression vectors were verified via Sanger sequencing using U6 promoter Fwd primer. 

### 2.3. Plasmids, Genomic DNA and Proteins Extraction

Plasmids were extracted from the transformed bacteria culture by using the Monarch Plasmid Miniprep Kit (New England Biolabs). The T47D and HEK293T cells genomic DNA were extracted by using the Monarch Genomic DNA Purification Kit (New England Biolabs). The digested plasmids and PCR amplicons were purified by using either the Monarch DNA Gel Extraction or Monarch PCR and DNA Cleanup Kits (New England Biolabs). All these steps were performed by following the manufacturer’s recommendations. The yield and quality of the prepared plasmid and genomic DNA were determined by using the NanoDrop™ 2000/2000c Spectrophotometers (Thermo Scientific™, Waltham, MA, USA). The proteins were extracted from the cells by using 1X RIPA buffer in the presence of 1:100 protease inhibitor cocktail (Nacalai Tesque, Kyoto, Japan). The extracted proteins were quantified by using the Pierce BCA Protein Assay Kit (Thermo Fisher, Waltham, MA, USA). 

### 2.4. Lentiviral Particles Production and Transduction

The HEK293T cells, seeded on the 6-well plate, were co-transfected with the mixture of packaging plasmids psPAX2 (1.3 μg) and pMD2.G (0.5 μg), and the plasmid of interest (1.5 μg) using Attractene transfection reagent (Qiagen, Hilden, Germany). The media containing the lentiviral particles was collected 72 h post-transfection and filtered through a MinisartNML 0.45 μM syringe filter (Sartorius, Göttingen, Germany), followed by either storage at −80 °C or use for transduction. For transduction, the media containing the lentiviral particles was added into the cells in the presence of 8 µg/mL polybrene (Merck Millipore, Burlington, MA, USA). The positively transduced cells were selected with 700 µg/mL Hygromycin B solution (Nacalai Tesque). 

### 2.5. TP53 Prime Editing: T47D Cells Transfection

The T47D cells were seeded onto 12-well plate. After reaching 70% confluency on the following day, the cells were co-transfected with the pCMV-PE2, *TP53* T > C pegRNA and its respective PE3 plasmids mixture by using the Attractene transfection reagent (Qiagen, Hilden, Germany) according to the manufacturer’s recommendations. The co-transfection was performed by testing two different conditions denoted as 1X and 3X. In brief, the 1X condition consisted of 900 ng pCMV-PE2, 300 ng *TP53* T > C pegRNA, and 100 ng PE3 plasmids. On the other hand, the 3X condition consisted of triple the amounts of each plasmid. The transfection was performed in serum- and antibiotic-free media, and this media was replaced with the complete media 24 h post-transfection. Genomic DNA was extracted from the pool of the transfected cells 7 days post-transfection, followed by PCR amplification of the corresponding *TP53* region and genotyping using Sanger sequencing. 

### 2.6. TP53/HEK3 Prime Editing: T47D and HEK293T Cell Electroporation 

The T47D cell electroporation was performed using the Neon^TM^ electroporation system in 100 μL Neon^TM^ Electroporation tip (Thermo Fisher). In brief, T47D or HEK293T cells, in suspension, were electroporated with a plasmids mixture containing 9 μg pCMV-PE2, 3 μg of either *TP53* pegRNA, or *HEK3* C > G pegRNA, and 1 μg of respective PE3 nicking plasmids. The electroporation was performed in serum- and antibiotic-free media, and the electroporation parameters used were 1700 V pulse voltage, 20 ms pulse width, and 1 pulse number. For the HEK293T cells, the following parameters were used: 1100 V pulse voltage, 20 ms pulse width, and 2 pulse number. The media was replaced with complete media 24 h post-electroporation. To establish the single cell-derived clones for *TP53* prime editing, the electroporated T47D and HEK293T cells were subjected to single cell dilution. The cells were seeded at a density of 1 cell/well on a 96-well plate and were allowed to grow for 3–4 weeks, with interval media replenishment. The formation of single cell-derived clones was monitored under the microscope and expanded for subsequent experiments. 

### 2.7. TP53 Genotyping Using Sanger Sequencing

The *TP53* region of interest was amplified from the genomic DNA of the transfected/electroporated cell pools or the single cell-derived clones. In brief, 25 ng of the genomic DNA was used as starting material, and the region of interest was amplified in the master mix consisting of 10 μM *TP53* PCR Fwd and 10 μM *TP53* PCR Rev primers, 0.25x AmpliTaq Gold™ DNA Polymerase, PCR Buffer II, MgCl_2,_ and dNTPs mix according to the manufacturer’s recommendations (Applied Biosystems, Waltham, MA, USA). The PCR amplicons were column purified and subjected to Sanger sequencing using 10 μM *TP53* PCR Fwd primer. 

### 2.8. Amplicon Targeted Sequencing

The *TP53* and *HEK3* region were amplified using Accuprime PFX Supermix and the region-specific PCR primer pairs. The PCR forward primer contains the i5 adapter sequence, and the reverse PCR primer contains the i7 adapter sequence (Supplementary Table S2). The DNA concentration was determined using a Qubit fluorometer (Thermo Fisher), and the DNA integrity was assessed using gel electrophoresis. The PCR product was purified using Monarch PCR and DNA Cleanup Kits (New England Biolabs) and subjected to Illumina sequencing library preparation. The amplicon libraries were run on Illumina MiSeq 300 (paired end). The sequencing raw reads were processed using BBDuk of the BBTools Packages (https://jgi.doe.gov/data-and-tools/bbtools/ (accessed on 1 April 2022). The prime editing efficiency was determined using the CRISPR RGEN Tools-PE Analyzer (http://www.rgenome.net/pe-analyzer/, accessed on 10 April 2022) according to the webtool instructions. Prime editing efficiency was calculated as a percentage (number of reads with desired edit/number of total aligned reads). For unwanted by-product analysis at the pegRNA and nicking sgRNA binding site, a comparison range (R) of 129 bp or 76 bp was used for the *HEK3* locus and the *TP53* gene, respectively. The frequency of by-products was calculated as a percentage (number of reads with undesired edit/number of total aligned reads).

### 2.9. Western Blotting

Briefly, 50 µg of the extracted proteins were separated using 10% SDS-PAGE and then transferred onto a nitrocellulose membrane (GE Healthcare, Chicago, IL, USA; Amersham, UK). The membrane was blocked for 1 h in 5% nonfat dry milk prepared in 0.1% Tween20- 1X Tris-buffer saline (TBS-T). The membrane was incubated with a primary antibody overnight at 4 °C. The membrane was washed with 0.1% TBS-T three times on the next day, followed by 1 h of secondary antibody incubation at room temperature. The membrane was washed three times with 0.1% TBS-T, and the signals were developed using SuperSignal™ West Pico PLUS Chemiluminescent Substrate (Thermo Fisher) and visualized using the ChemiDoc XRS+ Gel Imaging System (Bio-Rad, Hercules, CA, USA). The following primary antibodies and dilution in 5% nonfat dry milk were used: TP53 (Santa Cruz, Dallas, TX, USA, sc-126, 1:1000) and GAPDH (Thermo Fisher, #MA5-15738, 1:1000). The rabbit anti-mouse Ig/HRP secondary antibody (Dako, Santa Clara, CA, USA, P026002) was used at a dilution of 1:1000 in 5% nonfat dry milk.

## 3. Results

### 3.1. Verification of TP53 Mutation in T47D Breast Cancer Cells

**Figure 1:**
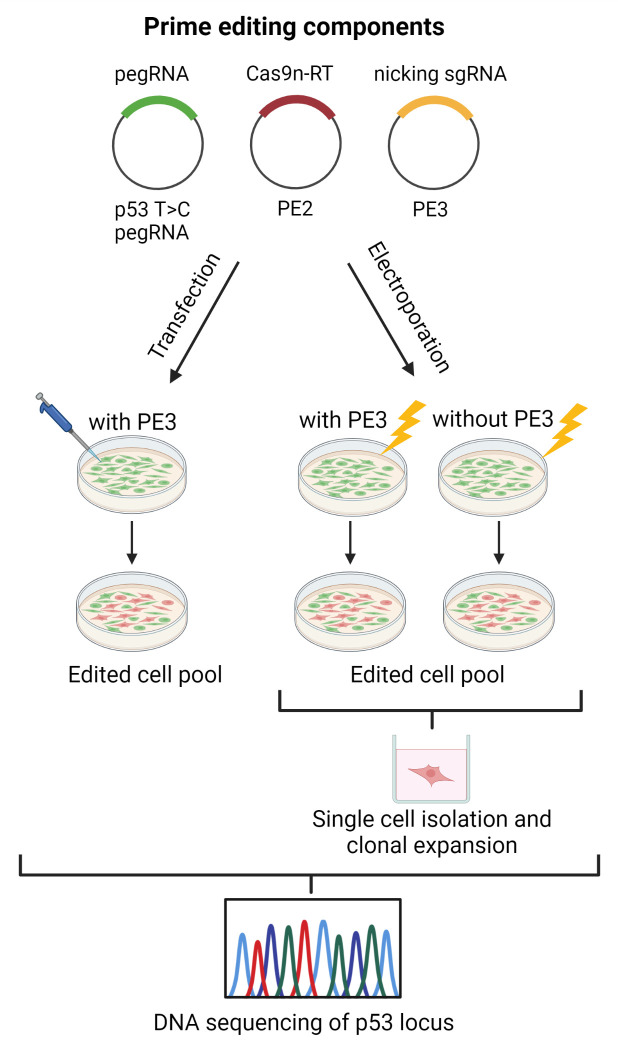
*TP53* T > C prime editing workflow in the T47D breast cancer cell line.

**Figure 2:**
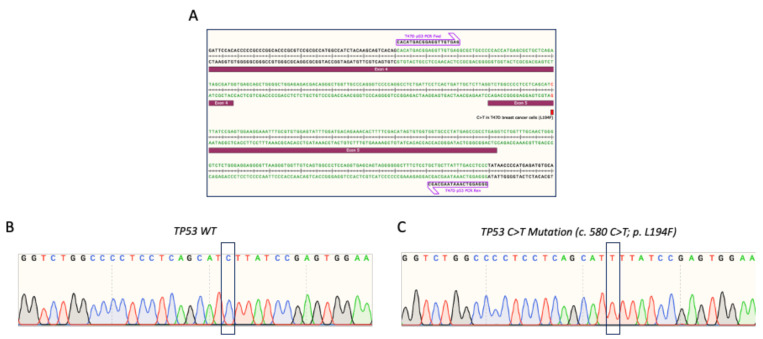
*TP53* c.580 C > T mutation characterization in T47D breast cancer cell line. (**A**) Schematic of the to-be-edited *TP53* region and the position of the C > T mutation. The schematic was generated using SnapGene Viewer software. (**B**,**C**) Sanger sequencing verification of the to-be-edited *TP53* region in (**B**) *TP53* wild-type HEK293T and (**C**) *TP53* mutated (c.580 C > T) T47D cells.

We referred to the guidelines recommended by Anzalone et al. [35] to establish our prime editing workflow for reverting the TP53 missense mutation in the T47D breast cancer cell line (Figure 1). According to the Sanger Institute and Ensembl databases, the T47D cells harbor a C > T missense mutation in the TP53 coding sequence at the 580 (c.580 C > T) position, which leads to the substitution of leucine to phenylalanine (p.L194F). We first verified the presence of this c.580 C > T mutation in the T47D cell line via Sanger sequencing. We amplified and sequenced the PCR amplicons of the TP53 region harboring this mutation from the T47D genomic DNA (Figure 2A). For comparison, we also sequenced this TP53 region wild-type sequence that was extracted from the HEK293T cells. We confirmed the presence of the TP53 homozygous cytosine to thymine substitution (c.580 C > T; p.L194F) in the T47D cells, but not in the HEK293T cells (Figure 2B,C).

### 3.2. pegRNAs and PE3 Plasmids Construction

**Figure 3:**
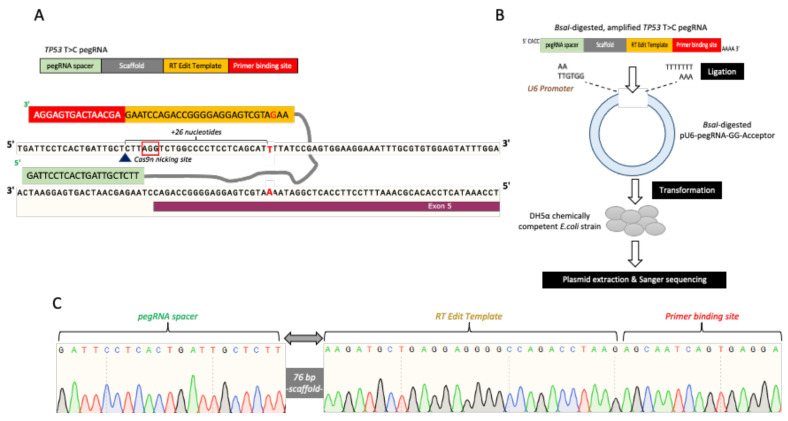
*TP53* T > C pegRNA plasmid construction. (**A**) Schematic of the *TP53* T > C pegRNA sequence and binding site at the to-be-edited *TP53* region. (**B**) *TP53* T > C pegRNA plasmid construction workflow. (**C**) Sanger sequencing verification of the ligated *TP53* T > C pegRNA in the pU6-pegRNA-GG-acceptor.

Next, we designed and constructed the TP53 prime editing gRNA, hereafter referred to as TP53 T > C pegRNA, by following Anzalone et al. recommended strategy [35]. Briefly, the TP53 T > C pegRNA consisted of the (i) pegRNA spacer, (ii) scaffold, (iii) reverse transcription (RT) edit template, and (iv) primer binding site (PBS) (Figure 3A). The length of the TP53 pegRNA spacer was 21 bp, which bound to the opposing strand preceding the “AGG” PAM sequence. The RT edit template was 28 bp and contained the template to revert the “T” mutation to the wild-type “C” nucleotide. This allowed the T > C editing to occur 26 bp downstream of the nicking site following the Cas9n activity on the top DNA strand (Figure 3A). The 15 bp PBS site served as the reverse transcription primer for synthesizing the edited strand complementary sequences. The amplified double-stranded TP53 T > C pegRNA was cloned into the BsaI-digested pU6-pegRNA-GG-acceptor plasmid (Figure 3B). Upon ligation and plasmids propagation, we confirmed the presence of TP53 T > C pegRNA within the expression plasmid via Sanger sequencing (Figure 3C).

In addition, we also attempted to perform a base substitution in the HEK293T cells using the prime editing approach. We aimed to introduce the C > T mutation at the position c.580 of the TP53 coding sequence in the HEK293T cells (similar to the TP53 mutation in the T47D cells). A similar approach as that outlined in Figure 1 was employed, with slight modifications. We only performed electroporation for the HEK293T prime editing, and a different pegRNA was designed, which was the TP53 C > T pegRNA. The TP53 C > T pegRNA design and cloning strategy was similar to the TP53 T > C pegRNA construction method described above. The presence of the ligated TP53 C > T pegRNA inside the pU6-pegRNA-GG-acceptor expression vector was confirmed via Sanger sequencing (Supplementary Figure S1). 

**Figure 4:**
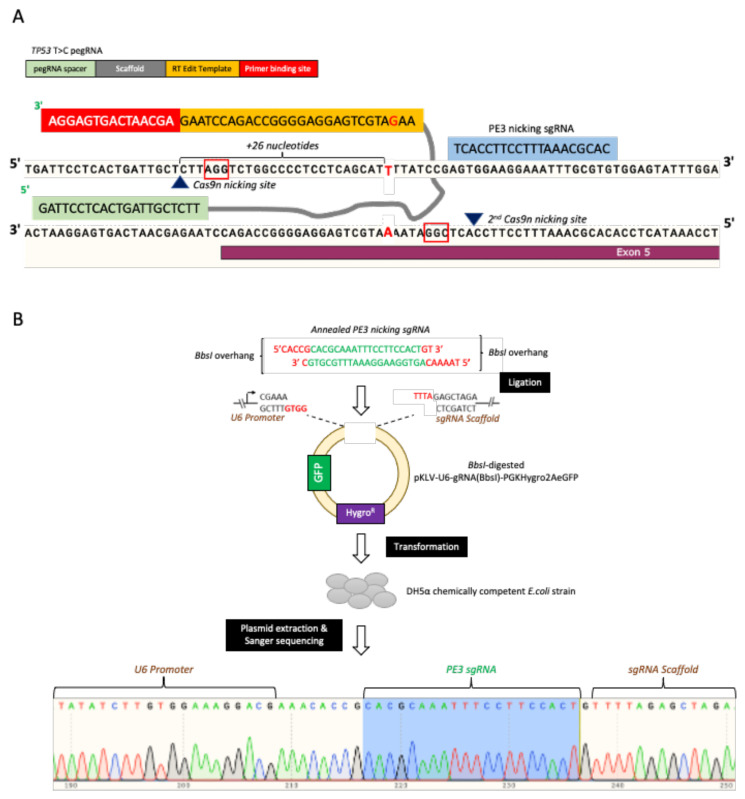
PE3 nicking sgRNA plasmid construction. (**A**) Schematic of the PE3 nicking sgRNA sequence and binding site at the to-be-edited *TP53* region. (**B**) PE3 nicking sgRNA plasmid construction workflow and the ligated sgRNA sequence verification.

It was suggested that the presence of an additional sgRNA, known as PE3, would enhance the prime editing efficiency. Therefore, we also designed and constructed this PE3 to introduce a second nicking site on the non-edited strand, as shown in Figure 4A. Standard sgRNA cloning was employed, where the annealed PE3 top and bottom strand was ligated into the BbsI-digested pKLV-U6-gRNA(BbsI)-PGKHygro2AeGFP expression vector. We confirmed the presence of the ligated PE3 sgRNA by Sanger sequencing (Figure 4B).

To evaluate our capacity to perform prime editing, we conducted a positive control experiment to induce a C > G substitution in the previously reported HEK3 locus [35,40] in the HEK293T and T47D cells. We utilized the same pegRNA, hereafter referred to as HEK3 C > G pegRNA, and respective PE3 nicking sgRNA as designed by Anzalone et al. [35]. The schematics of these constructs and the HEK3 C > G pegRNA cloning validation are shown in Supplementary Figure S2A,B. 

### 3.3. Assessing the Prime Editing Components Efficiency

Several strategies were employed to assess the activity or efficiency of the designed prime editing components prior to performing the experiments in the T47D and HEK293T cells. To test the TP53 T > C pegRNAs and PE3 targeting capabilities, we utilized the previously established T47D and MDA-MB-231 cells that stably expressing the wild-type Cas9. We have demonstrated in a parallel study that the wild-type Cas9 in these cells were efficient at introducing the double-strand break at the sgRNA binding site (data not shown). In this present study, we stably expressed PE3 in these T47D and MDA-MB-231 cells and performed TP53 Western blotting to check the expression of TP53 protein in these cells upon transduction with PE3. Moreover, we also transfected the TP53 T > C pegRNA into these Cas9/PE3-expressing T47D and MDA-MB-231 cells and performed the TP53 Western blotting. Compared to the control cells, we observed a reduction in the TP53 expression level in these T47D and MDA-MB-231 cells, expressing either PE3 alone or the combination of PE3 and TP53 T > C pegRNA (Supplementary Figure S3A,B). The reduction in TP53 expression indicated the double-strand break at the target region, followed by the introduction of INDELs that would result in a frameshift and/or nonsense mutations.

To check the Cas9n-RT PE2 activity, the PE2 plasmid was transfected into the HEK293T cells stably expressing two independent TP53-targeting sgRNAs, sgTP53_1, and sgTP53_2. These sgRNAs have been used in a parallel study and were confirmed to be efficient in targeting the TP53 in T47D and MDA-MB-231 cells (data not shown). The genomic DNA was extracted from the transfected HEK293T cells, and the region targeted by these sgTP53_1 and sgTP53_2 was amplified and subjected to Sanger sequencing. Interestingly, we found a large deletion between the sgRNAs binding sites (Supplementary Figure S4), which could be due to the PE2 nicking activity at the sgRNAs binding sites. Additionally, we also confirmed the efficiency of our transfection protocol in the T47D and HEK293T cells (Supplementary Figure S5).

### 3.4. T47D Cell Transfection and Assessment of TP53 Prime Editing Efficiency 

**Figure 5:**
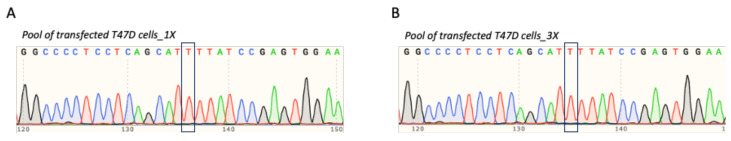
*TP53* T > C reversion efficiency in the pool of transfected T47D cells. (**A**,**B**) Sanger sequencing electropherogram of pool T47D cells transfected with (**A**) 1X and (**B**) 3X the amount of prime editing components.

As depicted in Figure 1, we co-delivered the prime editing components into the T47D cells, either via lipid-based transfection or electroporation. This was followed by assessing their respective editing efficiency, which was the reversion of the TP53 mutation at position c.580 from “T” to wild-type “C”. For the former approach, we co-transfected the T47D cells with either 1X or 3X amounts of the recommended prime editing components. We postulated that increasing the amount of transfected prime editing components would enhance the editing efficiency. We next checked the presence of “T” to “C” reversion in the transfected cell pool. If the prime editing strategy was efficient, as reported in some publications, we predicted that a significant fraction of the transfected cells would undergo the desired reversion. Hence, we expected to obtain a heterozygous “T/C” at the edited site due to the mixture of the edited and non-edited cells within the population. Unfortunately, we did not obtain the expected heterozygous “T/C” in either the pools of cells transfected with 1X or 3X the amount of prime editing components (Figure 5A,B).

### 3.5. T47D Cell Electroporation and Assessment of the TP53 Prime Editing Efficiency

**Figure 6:**
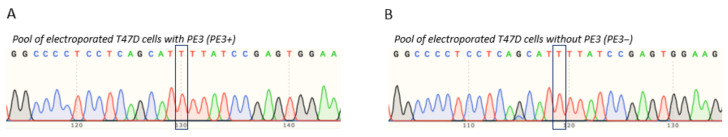
TP53 T > C reversion efficiency in the pool of electroporated T47D cells. (**A**,**B**) Sanger sequencing electropherogram of pool T47D cells electroporated with the prime editing components, either in the (**A**) presence or (**B**) absence of PE3 nicking sgRNA.

**Figure 7:**
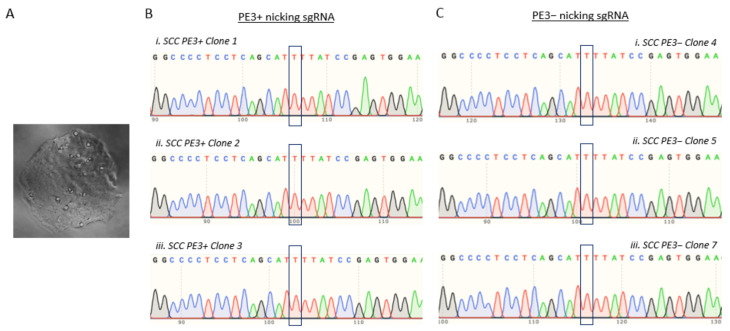
TP53 T > C reversion efficiency in the electroporated T47D single cell-derived clones. (**A**) Representative image of a single cell-derived clone from the PE3+ electroporated cells. (**B**,**C**) Sanger sequencing electropherogram of single cell-derived clones previously electroporated with prime editing components, either in the (**B**) presence or (**C**) absence of PE3 nicking sgRNA.

We inferred that the lipid-based transfection was possibly not efficient to deliver all of the prime editing components into T47D cells. Therefore, we also performed electroporation, since this approach has been proven to increase the uptake of exogenous plasmids. The T47D cells were co-electroporated with the prime editing components, either in the presence or absence of the PE3 plasmid, which hereafter is referred to as either PE3+ or PE3−, respectively. This was followed by assessing the “T” to “C” reversion efficiency in the pooled cells and single cell-derived clones setting (Figure 1). Instead of obtaining the postulated heterozygous “T/C”, we only obtained homozygous “T” for both the PE3+ and PE3− pooled cells (Figure 6A,B). In this regard, we presumed that most cells in the population were non-edited, possibly due to the low editing event and the fact that Sanger sequencing was not able to capture the successfully edited cells. Hence, we performed single cell isolation and clonal expansion to screen for single cell-derived clone cells that underwent successful “T” to “C” reversion (Figure 7A). To this end, we generated and screened 11 and 7 single cell-derived clones from the PE3+ and PE3− -electroporated cells, respectively. Unfortunately, none of these isolated single cell-derived clones harbored the desired TP53 c.580 “T” to “C” reversion (Figure 7B,C).

### 3.6. Introducing c.580 C > T Mutation in TP53 Gene in HEK293T Cells

**Figure 8:**
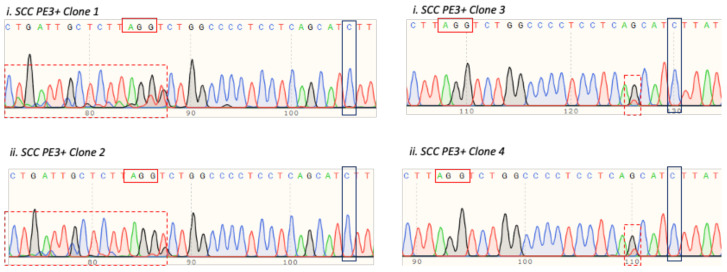
The TP53 C > T substitution efficiency of the electroporated HEK293T single cell-derived clones.

Moreover, we also performed the prime editing experiment in an additional system with the HEK293T cells. We attempted to substitute the “C” to “T” nucleotide at the TP53 c.580 position in these cells, which was the same as the TP53 mutation in the T47D cells. The HEK293T cells were co-electroporated with C > T pegRNA, PE3, and the PE2 plasmids, followed by single cell seeding and clonal expansion. The presence of the “C” to “T” mutation in the HEK293T single cell-derived clones was checked via Sanger sequencing, similar to the method previously described for the T47D cells. None of the screened HEK293T single cell-derived clones harbored the desired “C” to “T” substitution at the c.580 position. Nonetheless, we observed that two clones, namely clone 1 and clone 2, harbored overlap sequences in the proximity of the pegRNA spacer PAM site (Figure 8; left panel). These overlap sequences may possibly indicate the occurrence of PE2-mediated nicking at this region, followed by the introduction of INDELs upon repairing the nick. Moreover, we also identified two additional clones, clone 3 and 4, that showed similar G/T overlap upstream of the to-be-substituted “C” nucleotide (Figure 8; right panel). 

### 3.7. Assessing TP53 and HEK3 Primer Editing Using Amplicon Target Sequencing

**Figure 9:**
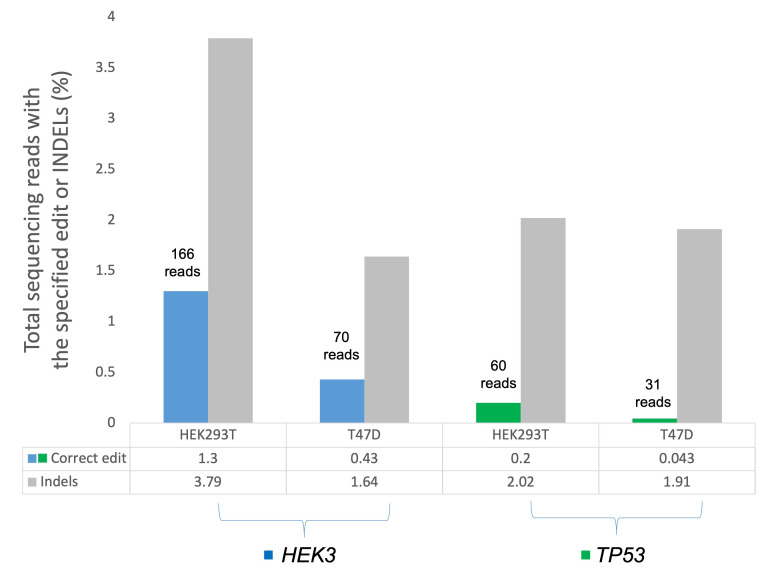
Frequency reads of the desired edits (%) at the *HEK3* and *TP53* target sites in the electroporated HEK293T and T47D cell pools using the amplicon target sequencing approach.

We postulated that the prime editing efficiency of these TP53 genes was low and therefore, could not be readily detected by the Sanger sequencing approach. To confirm whether there was any editing event present, we subjected the TP53 amplicons from the T47D and HEK293T cell pools to Illumina high-throughput sequencing. In parallel, we also ran the HEK3 amplicons from these two cell pools on the Illumina platform. The prime editing outcomes were evaluated in the electroporated cell population, without prior antibiotic selection or cells sorting. The amplicons target sequencing revealed there was the desired C > G substitution in the HEK3 target region in the HEK29T and T47D cells at the editing efficiency of 1.3% and 0.43%, respectively (Figure 9). For the TP53 prime editing, the desired substitution was also detected in the T47D and HEK293T cells, albeit the editing efficiency was significantly lower than in the HEK3 region. The editing efficiency for the HEK293T and T47D was 0.2% and 0.043%, respectively (Figure 9). Despite these lower editing efficiencies, it can be deduced that the prime editing events were indeed present in the cells. The amplicon target sequencing is far superior and sensitive for detecting the low editing efficiency, which might be missed in the traditional Sanger sequencing. 

## 4. Discussion

Prime editing has promising potential to become one of the key technologies for advancing the biological and medical fields. After the successful repurposing of its predecessor, CRISPR-Cas9, for eukaryotic gene editing, the scientific communities were intrigued by the potentials of this CRISPR-Cas9 technology. Due to its simplicity and flexibility, the CRISPR-Cas9 gene editing technology has been widely embraced and broadened to a wide variety of applications [26,36]. Clinically, the CRISPR-Cas9-based therapeutic approaches have been approved for cancer immunotherapy and treating blood disorders [28,30]. There are several other ongoing clinical trials that hope to reach the bedside in near future. Nonetheless, efficacy and safety issues related to off-target effects have hampered the efforts to fully exploit this CRISPR-Cas9 technology for clinical uses [32]. Therefore, the prime editing technology was developed with the aim to overcome these shortcomings and provide a more robust gene editing tool [37]. 

The CRISPR-Cas9 technology relies on either DSB-induced non-homologous end joining (NHEJ) or homology-directed repair (HDR) to perform the gene editing. This could incorporate random INDELs and induce undesired off-target effects. Moreover, the efficiency of HDR-mediated precise gene editing is considerably low because it requires a repair template, and the HDR itself is the less-favored repair mechanism [47,48]. In contrast, the prime editing technology does not rely on DSB and is also capable of introducing any desirable edits, indicating that it has promising therapeutic potential to correct numerous disease-causing mutations [49]. Since this technology is relatively new, researchers are currently attempting to improve the prime editing efficiency, which has been reported to be unsatisfactory in some experimental models. For example, Sürün et al. observed only a 3–6% successful editing rate in their prime-edited human induced pluripotent stem cells, while another study reported a substantially low editing efficiency to correct mutations in Alzheimer and Duchenne Muscular Dystrophy-related genes [39,43]. Geurts et al. also found that prime editing had an overall lower efficiency for repairing the cystic fibrosis CFTR-R785 mutation as compared to the base editing approach [44,50].

In this present study, we employed the prime editing tool to substitute a base in the *TP53* and *HEK3* gene in T47D and HEK293T cells. At first, the Sanger sequencing did not detect the desired edit in the *TP53* gene in either cell line. However, by employing the more sensitive Illumina-based amplicon targeted sequencing, we were able to detect the desired base substitutions in the *TP53* gene and *HEK3* locus in both cell lines. It is noteworthy to highlight that the observed editing efficiencies were significantly lower than what had been initially expected. Although we had confirmed the activities of the designed *TP53* pegRNA and PE3, and the Cas9n-RT PE2, there were still limitations with our prime editing strategy. In order for the prime editing to take place, the cells need to uptake and co-express all components simultaneously. We, however, could not assess whether the cells co-expressed these three plasmids due to a lack of fluorescent protein or antibiotic selection markers in pCMV-PE2 and pegRNA (pU6-pegRNA-GG-acceptor). Hence, it would be useful if these plasmids, or any similar plasmids constructed in the future, encoded for at least a fluorescent protein or antibiotic selection marker. This would allow for the selection and analysis of relevant cell populations. In addition, optimizing the plasmids delivery protocols, either via lipid-based transfection or electroporation, could increase the efficiency of plasmids uptake by the cells. It is also important to check the activity of the prime editing components prior to performing the experiments. For instance, as reported herein, the pegRNA or PE3 targeting efficiency could be assessed by expressing these plasmids in cells expressing the wild-type Cas9. This can be followed by performing Western blotting, assessing the cleavage using T7 endonuclease 1 assay, or Sanger sequencing, to check whether there is incorporation of INDELs at the expected cleavage site upstream of the PAM sequence. We believe that it is also worth developing quality control or “efficiency assessment” steps that are specific to this prime editing technology in the future.

On another note, we first performed Sanger sequencing to check the presence of the desired edits, but did not obtain positive results. This approach, however, was not optimal to detect an edit that has low efficiency, especially in the pooled cells setting. Moreover, if we proceed to single cell isolation, a large number of single cell-derived clones must be screened to find the clones that actually harbor the desired edit. Therefore, a more sensitive approach, such as high throughput sequencing, should be the method of choice for detecting the less effective editing event. Enriching the positively transfected/electroporated cells prior to performing the high throughput sequencing, either by antibiotic selection or sorting, could increase the percentage of editing events detected [51].

Another limitation is in the pegRNA design strategy. There are three main components in the pegRNA constructs, and to date, there is no definite consensus on the optimal length for each of these components. In this study, we relied solely on the one pegRNA construct for each model, which was designed according to the recommendations of Anzalone et al. [35]. Our T > C/C > T *TP53* pegRNA consisted of a 21 bp pegRNA spacer, a 28 bp RT edit template, and a 15 bp PBS. Ideally several pegRNA constructs, varied in their component length, should have been tested for their targeting and editing efficiency. Unfortunately, we did not have much flexibility to optimize our pegRNA design for editing this specific single nucleotide in the *TP53* gene. In fact, the pegRNA designs have been the drawback in achieving favorable editing efficiency. Several recent studies have been focused on optimizing the length of the pegRNA components to improve editing efficiency [38,39,40,42,52]. For instance, Kim et al. found the optimal lengths for the RT edit template and PBS were 12 bp and 13 bp, respectively [42]. Ideally, the PBS should be GC-rich, and the RT edit template should end with “G,” if it is less than 12 bp [42]. Park et al. also tested various lengths for the RT edit template (10–18 bp) and PBS (814 bp) length [53]. Interestingly, they found that the introduction of additional components, namely the proximal dead sgRNA and chromatin-modulating peptides, significantly improved the editing efficiency of mouse embryonic cells [53]. Another study incorporated a nuclear localization signal (NLS) in the PE2 vector, referred to as PE2*, and found that this PE2* variant enhanced the editing efficiency as compared to PE2 [54]. 

Despite these recommendations, the pegRNAs still need to be designed and optimized based on the to-be-edited region. This is due to the differences in genomic architecture and the availability of the editing features, such as PAM sequence. Because of these wide-ranging parameters, the pegRNAs optimization process could be laborious and potentially less practical for those who have limited resources. To overcome this, several web tools have been developed, namely PrimeDesign, Pegfinder, and CRISPR RGEN, to facilitate the designing of optimal pegRNAs for prime editing experiments [55,56,57]. Interestingly, the CRISPR RGEN and another webtool, CRISPResso2, can be utilized to analyze prime editing efficiency [57,58]. These webtools are indeed valuable additions to prime editing research. Hopefully, this could pave the way for the development of more robust pegRNA designing and prime editing analysis webtools that would transform the prime editing field. Overall, the prime editing tool is still in its early years, and there is definitely more room for further optimizations and advancements. However, it still has several advantages and holds huge therapeutic potential when compared to the CRISPR-Cas9 gene editing technology. The insights gained from our study and others will be valuable for improving the efficiency of this fledgling prime editing technology. 

## Data Availability

Not applicable.

## Supplementary Materials

The following are available online at https://www.mdpi.com/article/10.3390/cells11101612/s1, Table S1: pegRNA and sgRNA sequences; Table S2: Primer sequences. Figure S1: Sanger sequencing verification of the ligated *TP53* C > T pegRNA in the pU6-pegRNA-GG-acceptor; Figure S2: *HEK3* C > G pegRNA and PE3. (**A**) Schematic of *HEK3* C > G pegRNA and PE3 sequence and their respective binding sites at the *HEK3* region. (**B**) Sanger sequencing verification of the ligated *HEK3* C > G pegRNA in the pU6-pegRNA-GG-acceptor; Figure S3: Assessment of *TP53* T > C pegRNA and PE3 targeting efficiency. (**A**,**B**) The expression of TP53 protein in the (**A**) T47D and (**B**) MDA-MB-231 cells that expressed the wild-type Cas9 along with the indicated plasmids; Figure S4: Assessment of PE2 nicking activity in the HEK293T cells stably expressing sgTP53_1 and sgTP53_2. The respective location of the Sanger sequencing results within the *TP53* genomic locus is shown by the red and green boxes. There was a large genomic region deletion between the sgTP53_1 cut site (**red box**) and around 90 base pairs upstream of the sgTP53_2 PAM sequence (**green box**); Figure S5: Transfection protocol efficiency assessment. (**A**,**B**) Representative images of eGFP^+^ (**A**) HEK293T and (**B**) T47D cells transfected with eGFP-encoded PE3 plasmid. (10× magnification.)
