# Supplementary material for: Reverting TP53 Mutation in Breast Cancer Cells: Prime Editing Workflow and Technical Considerations
Source: Cells. 2022 May 11;11(10):1612. doi: 10.3390/cells11101612 (PMC9139850; doi:10.3390/cells11101612)
Supplement: Supplementary file 1 [file cells-11-01612-s001.zip › cells-1406916-supplementary.pdf]

Supplementary Table S1. pegRNA and sgRNA sequences.

Construct	Sequence (5′-3′)
TP53 T>C pegRNA	ATCGGGTCTCACACCGATTCCTCACTGATTGCTCTTGTTTTAGAGCTAGAAATA GCAAGTTAAAATAAGGCTAGTCCGTTATCAACTTGAAAAAGTGGCACCGAGTC GGTGCAAGATGCTGAGGAGGGGCCAGACCTAAGAGCAATCAGTGAGGATTTTA GAGACCCGAT
TP53 C>T pegRNA	ATCGGGTCTCACACCGATTCCTCACTGATTGCTCTTGTTTTAGAGCTAGAAATA GCAAGTTAAAATAAGGCTAGTCCGTTATCAACTTGAAAAAGTGGCACCGAGTC GGTGCAAAATGCTGAGGAGGGGCCAGACCTAAGAGCAATCAGTGAGGATTTTA GAGACCCGAT
HEK C>G pegRNA	ATCGGGTCTCACACCGGCCCAGACTGAGCACGTGAGTTTTAGAGCTAGAAATAGC AAGTTAAAATAAGGCTAGTCCGTTATCAACTTGAAAAAGTGGGACCGAGTCGGTC CTGGAGGAACCAGGGCTTCCTTTCCTCTGCCATCACGTGCTCAGTCTGTTTTAGAG ACCCGAT
Top *TP53* nicking sgRNA	CACCGCACGCAAATTTCCTTCCACTGT
Bottom *TP53* nicking sgRNA	TAAAACAGTGGAAGGAAATTTGCGTGC
Top *HEK3+90* nicking sgRNA	CACCGGTCAACCAGTATCCCGGTGCGT
Bottom *HEK3+90* nicking sgRNA	TAAAACGCACCGGGATACTGGTTGACC

Supplementary Table S2. Primer sequences.

Construct	Sequence (5′-3′)
pegRNA PCR Fwd	ATCGGGTCTCACACC
pegRNA PCR Rev	ATCGGGTCTCTAAAA
TP53 PCR Fwd	CACATGACGGAGGTTGTGAG
TP53 PCR Rev	GGGAGGTCAAATAAGCAGCA
HEK3 PCR Fwd	ATGTGGGCTGCCTAGAAAGG
HEK3 PCR Rev	GGTGCTGAAAGCCACTGGGC
U6 promoter Fwd	GAGGGCCTATTTCCCATGATTCC
i5 Fwd adapter	TCGTCGGCAGCGTCAGATGTGTATAAGAGACAG
i7 Rev adapter	GTCTCGTGGGCTCGGAGATGTGTATAAGAGACAG

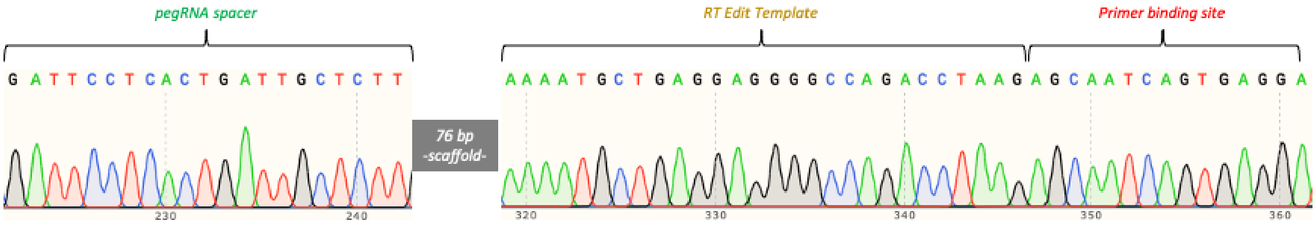

Supplementary Figure S1. Sanger sequencing verification of the ligated *TP53* C>T pegRNA in the pU6-pegRNA-GG-acceptor.

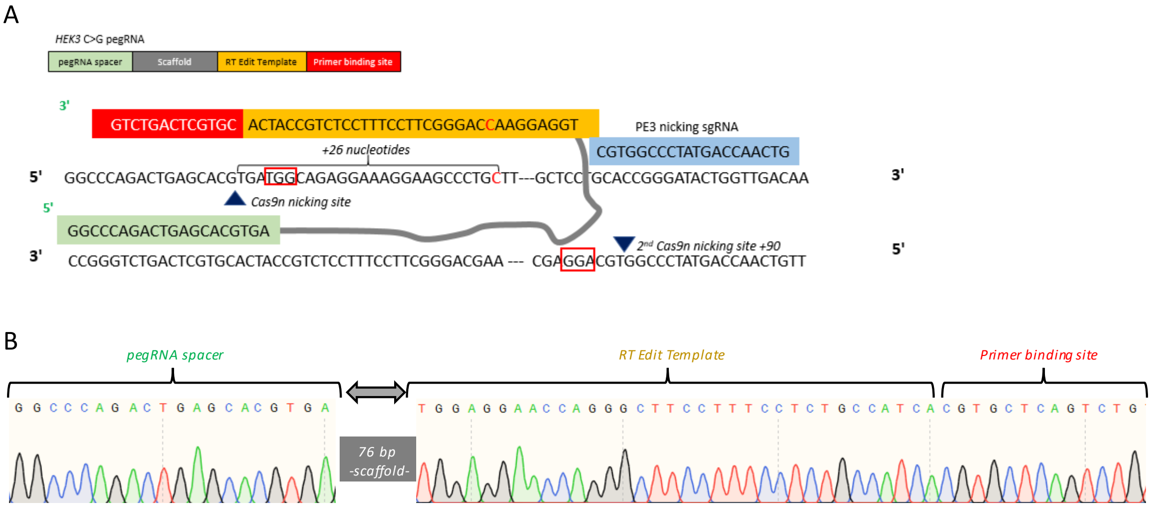

Supplementary Figure S2. *HEK3* C>G pegRNA and PE3. (**A**) Schematic of *HEK3* C>G pegRNA and PE3 sequence and their respective binding sites at the *HEK3* region. (**B**) Sanger sequencing verification of the ligated *HEK3* C>G pegRNA in the pU6-pegRNA-GG-acceptor.

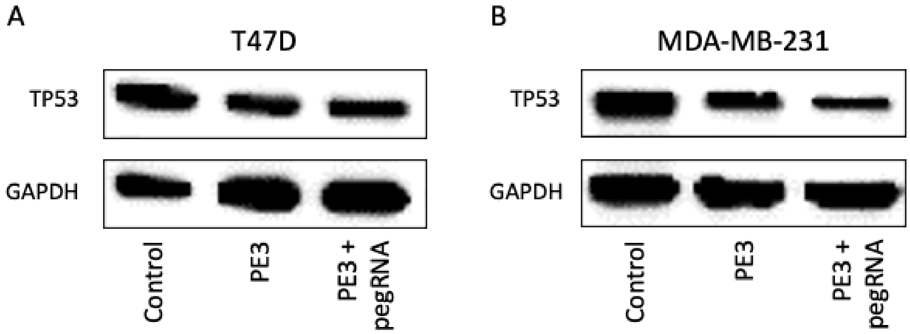

Supplementary Figure S3. Assessment of *TP53* T>C pegRNA and PE3 targeting efficiency. (**A**,**B**) The expression of TP53 protein in the (**A**) T47D and (**B**) MDA-MB-231 cells that expressed the wild-type Cas9 along with the indicated plasmids.

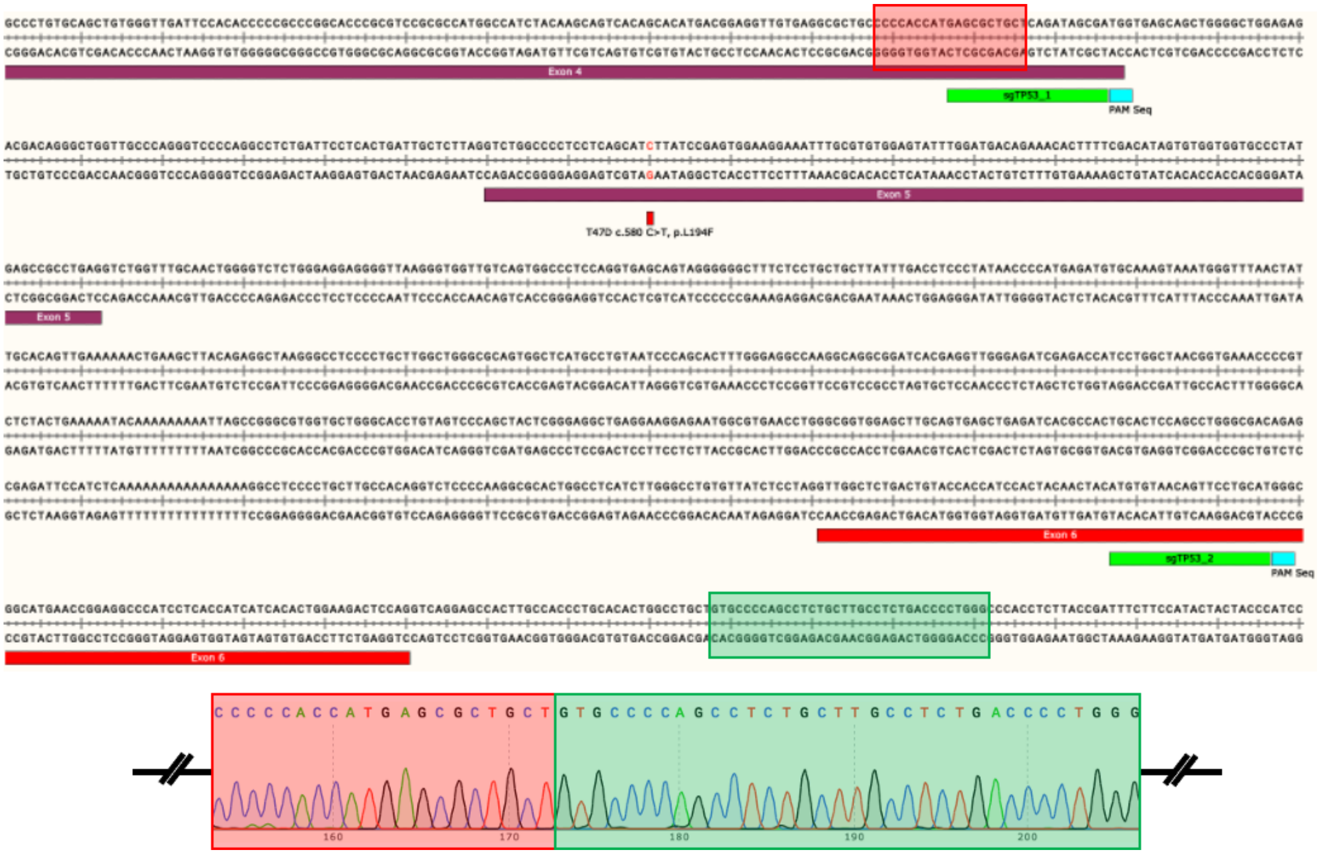

Supplementary Figure S4. Assessment of PE2 nicking activity in the HEK293T cells stably expressing sgTP53_1 and sgTP53_2. The respective location of the Sanger sequencing results within the *TP53* genomic locus is shown by the red and green boxes. There was a large genomic region deletion between the sgTP53_1 cut site (**red box**) and around 90 base pairs upstream of the sgTP53_2 PAM sequence (**green box**).

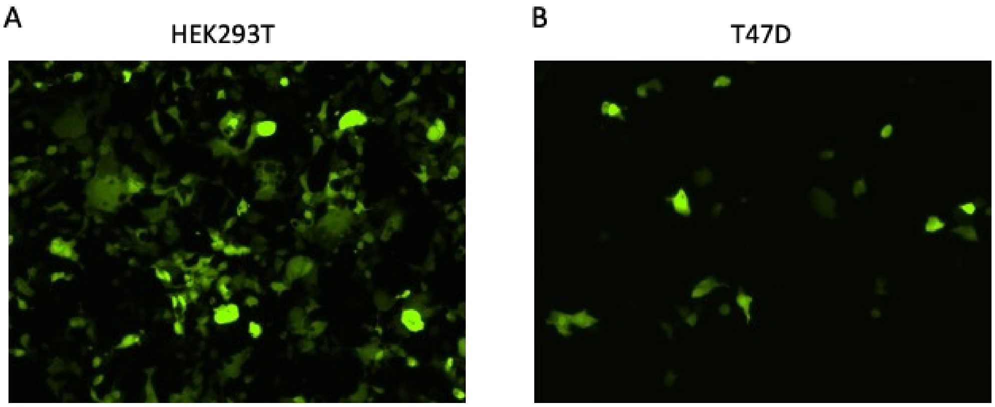

Supplementary Figure S5. Transfection protocol efficiency assessment. (**A**,**B**) Representative images of eGFP^{+} (**A**) HEK293T and (**B**) T47D cells transfected with eGFP-encoded PE3 plasmid. (10X magnification.)
